# The Complement System: A Prey of *Trypanosoma cruzi*

**DOI:** 10.3389/fmicb.2017.00607

**Published:** 2017-04-20

**Authors:** Kárita C. F. Lidani, Lorena Bavia, Altair R. Ambrosio, Iara J. de Messias-Reason

**Affiliations:** Laboratory of Molecular Immunopathology, Clinical Hospital, Federal University of ParanáCuritiba, Brazil

**Keywords:** *Trypanosoma cruzi*, complement system, complement regulatory proteins, evasion mechanism, innate immunity

## Abstract

*Trypanosoma cruzi* is a protozoan parasite known to cause Chagas disease (CD), a neglected sickness that affects around 6–8 million people worldwide. Originally, CD was mainly found in Latin America but more recently, it has been spread to countries in North America, Asia, and Europe due the international migration from endemic areas. Thus, at present CD represents an important concern of global public health. Most of individuals that are infected by *T. cruzi* may remain in asymptomatic form all lifelong, but up to 40% of them will develop cardiomyopathy, digestive mega syndromes, or both. The interaction between the *T. cruzi* infective forms and host-related immune factors represents a key point for a better understanding of the physiopathology of CD. In this context, the complement, as one of the first line of host defense against infection was shown to play an important role in recognizing *T. cruzi* metacyclic trypomastigotes and in controlling parasite invasion. The complement consists of at least 35 or more plasma proteins and cell surface receptors/regulators, which can be activated by three pathways: classical (CP), lectin (LP), and alternative (AP). The CP and LP are mainly initiated by immune complexes or pathogen-associated molecular patterns (PAMPs), respectively, whereas AP is spontaneously activated by hydrolysis of C3. Once activated, several relevant complement functions are generated which include opsonization and phagocytosis of particles or microorganisms and cell lysis. An important step during *T. cruzi* infection is when intracellular trypomastigotes are release to bloodstream where they may be target by complement. Nevertheless, the parasite uses a sequence of events in order to escape from complement-mediated lysis. In fact, several *T. cruzi* molecules are known to interfere in the initiation of all three pathways and in the assembly of C3 convertase, a key step in the activation of complement. Moreover, *T. cruzi* promotes secretion of plasma membrane-derived vesicles from host cells, which prevent the activity of C3 convertase C4b2a and thereby may hinder complement. In this review, we aim to present an overview on the strategies used by *T. cruzi* in order to circumvent the activation of complement and, consequently, its biological effects.

## Introduction

*Trypanosoma cruzi* is a hemoflagellate parasite of the order Kinetoplastida and Trypanosomatidae family (Levine et al., 1980) that causes CD. The parasite presents complex mechanisms of surveillance in the mammalian host and exerts direct influence on the course of CD (Watanabe Costa et al., 2016). CD is responsible for more expressive morbimortality than any other parasitic disease (World Health Organization [WHO], 2010; Bonney, 2014), resulting in a global annual burden of $627.5 million in health-care costs (Lee et al., 2013). It is estimated that 6–8 million people are infected with *T. cruzi* from 21 countries in Latin America (Stanaway and Roth, 2015), where 25 million people live at risk of acquiring the disease (Pereira and Navarro, 2013; World Health Organization [WHO], 2015). Furthermore, owing to the widespread human migration from CD-endemic areas, the disease has become an emerging global health concern, affecting several countries in Europe (World Health Organization [WHO], 2009; Navarro et al., 2012; European Centre for Disease Prevention and Control [ECDC], 2014), the United States (Bern and Montgomery, 2009), and Japan (Schmunis, 2007), where transmission occurs mainly through blood transfusions, organ transplants, or by congenital routes (Singh and Sehgal, 2010).

Although most of *T. cruzi-*infected people remain asymptomatic throughout their lives, the parasite-host interaction seems to be crucial for the development of the disease and the severity of the chronic symptomatic forms. In this context, the complement system plays an important role as a first line of host immune defense promoting the recognition, opsonization, and direct lysis of invading pathogens. However, during *T. cruzi* infection in humans, the activation of complement may present a dual role in both the acute and chronic phases of CD, initially being crucial in controlling the parasitemia, but later in the chronic phase contributing to the development or severity of the symptomatic forms because of its proinflammatory effect (Boldt et al., 2011; Weitzel et al., 2012; Luz et al., 2013, 2016). Considering that activation of complement by the lectin, classical, and APs leads to a proteolytic cascade and ultimately to a powerful lytic effect, this system is a special target for evasion strategies used by microbes in order to ensure infection success and possibly progression to chronic disease (Lambris et al., 2008). In fact, *T. cruzi* displays a range of different strategies to circumvent the harmful effects of the complement proteolytic cascade, which enables the parasite’s survival and development of CD. Therefore, we review here published information on *T. cruzi-*derived proteins that are involved in complement evasion which are critical to successful infection and disease progression.

## *Trypanosoma cruzi* and Chagas Disease

### *T. cruzi* Life Cycle

*Trypanosoma cruzi* infection occurs predominantly via vectorial transmission by triatomine bugs of the genera *Triatoma, Rhodnius*, and *Panstrongylus.* These bugs suck the blood of vertebrates infected with trypomastigotes, and this initiates the parasite life cycle. Once ingested by the insect vector, trypomastigotes are transformed in the anterior midgut either into spheromastigote or epimastigote forms. Epimastigotes multiply in the midgut and attach to the perimicrovillar membranes of the triatomine intestinal cells. At the most posterior region of the vector’s intestine and at the rectum, many epimastigotes detach from the intestinal surface and become infective metacyclic trypomastigotes, which are then released with feces and urine during blood meals. However, metacyclic trypomastigotes are not able to penetrate the intact host skin and enter through the rubbing or scratching of the bite wound, or through permissive mucosal or conjunctival surfaces at the inoculation site. Recruitment and fusion of the host lysosome with the parasite involves the formation of a parasitophorous endocytic vacuole, which is needed for parasite invasion of fibroblasts, macrophages, and epithelial cells (Souza et al., 2010; Nogueira et al., 2015). Subsequently, the parasite promotes enzymatic lysis of the vacuole membrane, differentiating into intracellular amastigotes, which after nine cycles of binary division become trypomastigotes that are eventually released into the circulation as bloodstream trypomastigotes (Dvorak and Hyde, 1973; Dvorak and Howe, 1976). These either infect new cells or are taken up by a new insect vector during a blood meal, repeating the parasite cycle.

**FIGURE 1:**
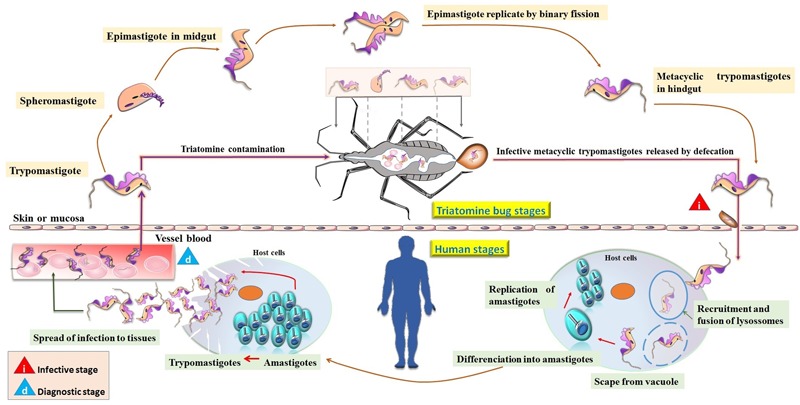
**Life cycle of *Trypanosoma cruzi*.** Transmission is initiated by insect vectors that defecate after a blood meal and release metacyclic trypomastigotes near the bite wound. This infective stage is characterized by the invasion of host cells by trypomastigotes forming the parasitophorous vacuole, from which they subsequently escape, differentiate into amastigotes, and replicate in the cytosol. The amastigotes then divide, differentiate into trypomastigotes, and upon rupture of the cell spread the infection to tissues. Trypomastigotes reach the bloodstream, where they are eventually taken up by the insect vector or infect new cells. In the triatomine bugs, trypomastigotes differentiate into spheromastigotes becoming initially short epimastigotes (mid-log). After migration to the bug’s hindgut, elongate epimastigotes (late-log) attach to the waxy gut cuticle and give rise to infectious metacyclic trypomastigotes, completing the parasite life cycle.

Once in the bloodstream of a mammalian host, *T. cruzi* is able to infect several cell types from a variety of tissues thereby spreading to new infection sites where they differentiate into intracellular amastigotes (Watanabe Costa et al., 2016) (**Figure 1**). Besides vectorial transmission, *T. cruzi* can also be transmitted through blood transfusion, organ transplantation, transplacentally, the ingestion of contaminated food or drinks, or by accidental exposure; however, the physiopathological mechanisms of such transmissions are still unclear.

### Chagas Disease

Several clinical manifestations may result in humans from *T. cruzi* infection. This initiates with an acute phase that last for about 2 months characterized by high parasitemia. In this stage, the diagnosis may be achieved by direct visualization of the parasite in blood and by the detection of IgG antibodies against *T. cruzi* antigens. Although most acute cases are oligosymptomatic or asymptomatic, initial dermatologic manifestations resulting in a skin lesion (chagoma), eyelid edema and conjunctivitis (Romaña sign), or generalized morbilliform eruption (schizotrypanides) may be present (Nunes et al., 2013). Other symptoms may include anorexia, fever, headache, dyspnea, abdominal pain, cough, hepatosplenomegaly, rash, painful nodules, generalized body swelling, and myocarditis (Hidron et al., 2010; Malik et al., 2015).

Following the acute phase, most infected people enter into a prolonged asymptomatic indeterminate form of the chronic disease and will never develop Chagas-related symptoms. However, after 10–30 years of infection approximately 30–40% of chronically infected people will present some clinical manifestations such as cardiomyopathy (20–30%), digestive commitment (10–15%) or both (1–5%) (Rassi and Marin-Neto, 2010). Chronic cardiomyopathy represents the most severe and life-threatening manifestation of human CD, ranging from asymptomatic electrocardiogram abnormalities to congestive heart failure, arrhythmias, and/or thromboembolic events (Biolo et al., 2010) that are associated with high morbidity and mortality. In fact, mortality related with CD is generally due to cardiovascular involvement, with approximately 12,000 deaths every year (Salvatella et al., 2013), with sudden death accounting for around 60%, heart failure 25–30% and stroke 10–15% (Rassi et al., 2001).

**FIGURE 2:**
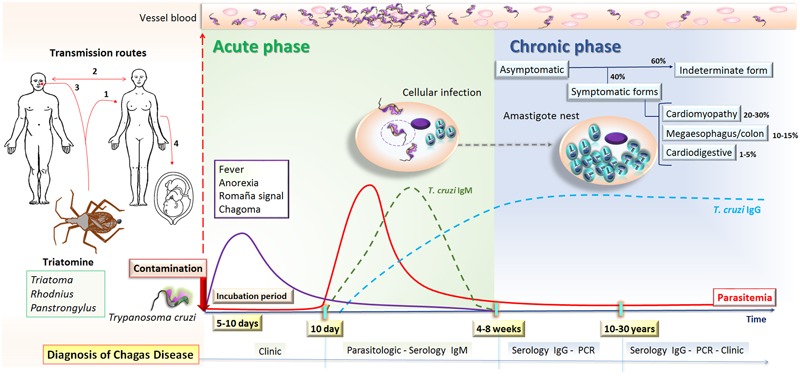
**Natural course of human *T. cruzi* infection.** *T. cruzi* transmission can occur by (1) vectorial (2) blood transfusion or organ transplantation, (3) oral, or (4) congenital routes. The incubation period lasts for 5–10 days after human contamination with *T. cruzi* from triatomines, which is followed by the acute phase of Chagas disease that lasts 4–8 weeks. This phase is characterized by circulating trypomastigotes, which can be visualized in the blood and IgM anti-*T. cruzi* antibodies can be detected after 10 days of infection. Most patients have non-specific symptoms, such as fever and anorexia, or are asymptomatic, and may develop inflammation and swelling at the site of inoculation in the skin or conjunctiva, characterizing chagoma and Romaña’s signal, respectively. The chronic phase begins once parasitemia falls below detectable levels by microscopy, usually 4 to 8 weeks after the onset of infection, and therefore diagnosis is based on the detection of anti-*T. cruzi* IgG antibodies or molecular tests. In this phase, most infected people enter a prolonged asymptomatic form known as the indeterminate form and will never develop Chagas-related symptoms. However, after 10–30 years around 30–40% of chronically infected people will present some clinical manifestations including cardiac, digestive, or cardiodigestive complaints.

Standard approaches for CD diagnosis in the chronic phase require at least two different serological tests, generally using ELISA and IFA (Bern et al., 2007). Additionally, amplification of parasite DNA by PCR may be employed in some uncertain cases (Schijman et al., 2011) (**Figure 2**).

## The Complement System

The complement system constitutes a key component of innate immunity with a significant role in the first line of defense against invading microbes. It comprises more than 35 plasma and cell membrane receptor/regulator proteins that become activated mainly by three pathways: lectin (LP), classical (CP), and alternative (AP), resulting in important biological responses such as inflammation, phagocytosis, and lysis of pathogens (Ricklin et al., 2010). Activation of complement and its potent inflammatory response through the production of molecules with anaphylatoxin activity was first demonstrated by Dias da Silva and Lepow (1967). The functional role in vascular permeability, histamine release from mast cells and contraction of smooth muscle of the anaphylatoxins (C4a, C3a, and C5a) was subsequently reported (Dias da Silva and Lepow, 1967; Dias da Silva et al., 1967; Cochrane and Müller-Eberhard, 1968). Besides inflammatory response, the complement also plays an important role in the solubilization and removal of circulating immune complexes to avoid their deposition, which could result in tissue injury (Miller and Nussenzweig, 1974). In addition, the complement system links the innate and acquired responses through the activation of B lymphocytes and synthesis of immunoglobulins (Walport, 2001; Ricklin et al., 2010, 2016). Furthermore, complement is involved in the opsonization of apoptotic cells contributing to their phagocytosis and removal from circulation (Flierman and Daha, 2007).

Complement is activated mainly by three pathways, LP, CP, and AP, which lead to the generation of effector molecules, self-amplification, and the induction of immune signaling (Ricklin et al., 2010). The LP can be triggered through the binding of PRMs, such as MBL, ficolins (Ficolin-1 [or M-ficolin], Ficolin-2 [or L-ficolin], and Ficolin-3 [or H-ficolin]) and CL-K1, to PAMPs on the pathogen’s surface (Beltrame et al., 2015). Whereas carbohydrate-recognition domains in the MBL molecule bind to sugar moieties on the pathogen’s surface (such as D-mannose, glucose, L-fucose, and GlcNAc) (Weis et al., 1992), the three human ficolins bind to PAMPs by fibrinogen-like recognition domains and exhibit differences in their binding specificities (Gout et al., 2010). For instances, Ficolin-1 recognizes *N*-acetylated compounds (such as GlcNAc and *N*-acetylgalactosamine [GalNAc]) (Frederiksen et al., 2005), *O*-acetylated and glycan compounds containing sialic acid (Gout et al., 2010). Ficolin-2 also recognizes *N*-acetylated compounds and capsulated strains of bacteria (Frederiksen et al., 2005; Gout et al., 2010), while Ficolin-3 binds GalNAc, GlcNAc, D-fucose as mono/oligosaccharides and lipopolysaccharides (Sugimoto et al., 1998). In addition, CL-K1 senses mannose and fucose-containing microbial derived products (Keshi et al., 2006). Following the binding of MBL, ficolins, or CL-K1 to PAMPs, the LP is initiated by the activation of MASP-1 and MASP-2 resulting in active forms (Kjaer et al., 2013) that cleave C4 in C4a and C4b, and C2 in C2a and C2b, culminating with the formation of the LP C3 convertase (C4b2a) and C5 convertase (C4bC2aC3b) (Walport, 2001; Ricklin et al., 2010; Merle et al., 2015).

The activation of the CP depends mainly on the interaction of C1 with antigen-antibody complexes or alternatively, by PAMPs (lipopolysaccharides and porins from Gram-negative bacteria), phospholipids, apoptotic cells (phosphatidylserine) or pentraxins (C-reactive protein and pentraxin 3), among others (Alberti et al., 1993; Kishore et al., 2004). The C1 complex is formed of one C1q molecule and two molecules each of C1r and C1s (C1qC1r2C1s2). C1q initiates the activation of the CP by binding to CH3 or CH2 Fc domains of IgM and IgG, respectively, inducing a conformational change in C1q. This leads to the activation of C1r and C1s, and serine proteases that cleave C4 and C2 (Lörincz et al., 2000), forming the CP C3 and C5 convertases (C4b2a and C4bC2aC3b), similar to those generated in the LP (Ricklin et al., 2010).

The activation of the AP starts with spontaneous hydrolysis of the thiol-ester bond in C3 α-chain generating C3(H2O). This molecule exhibits a reactive site for the plasma protein FB, forming the complex C3(H2O)B. In this condition, FB can be cleaved by FD in Ba and Bb. The Bb fragment remains bound to C3(H2O), forming the first C3 convertase of AP (C3(H2O)Bb), which now exhibits serine protease activity cleaving further C3 molecules into C3a and C3b. Like C3(H2O), C3b exhibits reactive sites for FB binding allowing its cleavage by FD, resulting in the second C3 convertase of this pathway, C3bBb. Additionally, C3 binds to C3bBb forming C3bBbC3b, a complex with C5 convertase activity (Walport, 2001; Ricklin et al., 2010).

Once all three pathways are activated, the C5 convertases formed by both CP/LP (C4b2a3b) and AP (C3bBb3b) cleave C5 in C5a and C5b. The fragment C5b binds to C6, forming a stable complex C5b6, which recruits C7 resulting in a hydrophobic complex that targets cell membranes (mC5b-7). After C8 is incorporated to mC5b-7, the C5b678 complex becomes inserted in the cell membrane. Then, 12–18 copies of the C9 molecule polymerize forming the MAC. This pore-forming ring structure (C5b678(9)n) gets inserted into the cell as a transmembrane channel, favoring ionic imbalance and an increase in intracellular volume leading to membrane cell disruption (Kondos et al., 2010).

Since undesirable activation of complement may lead to inflammation and tissue damage, an effective and accurate system of regulatory molecules is required for the maintenance of its homeostasis. In this context, a variety of membrane-bound inhibitory proteins (such as CR1, CD59, CD46, and DAF or CD55) downregulate local complement activation, protecting host cells from unwanted complement lysis (Carroll and Sim, 2011; Noris and Remuzzi, 2013). In addition, several plasma regulator proteins (such as Factor H, Factor I, C1-INH, C4BP, and vitronectin) control soluble activated complement components and complexes (Carroll and Sim, 2011; Noris and Remuzzi, 2013). In general, either excessive or defective complement activation may be implicated in the pathogenesis of some conditions including autoimmune, chronic inflammatory, and infectious diseases (Ricklin and Lambris, 2013).

### Complement Activation by *T. cruzi*

**FIGURE 3:**
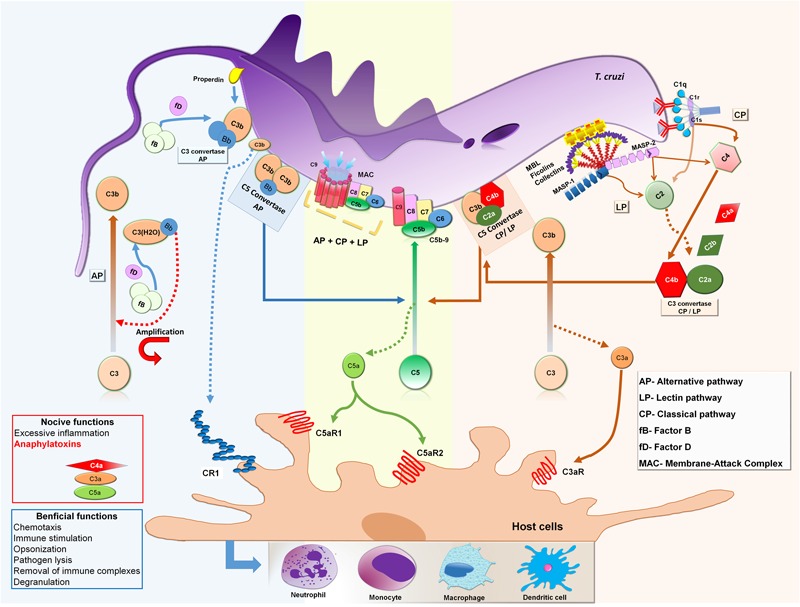
**Activation of complement system by *Trypanosoma cruzi* epimastigote forms.** MBL and ficolins recognize and bind to glycoproteins and carbohydrates present on the *T. cruzi* epimastigote surface initiating the LP. This interaction leads to conformational changes in MBL and ficolins and activation of MASP-1 followed by MASP-2 which cleavages C4 into C4a and C4b and C2 into C2a and C2b, generating C3 convertase (C4b2a). In presence of specific anti-*T. cruzi* antibodies, C1q molecules recognize and bind to IgM and IgG on the parasite’s surface initiating the CP. Subsequently, C1q suffers conformational changes and activates C1r, which cleaves and activates C1s that subsequently cleaves C4 and C2 generating C3 convertase (C4b2a), as in the LP convertase. When C3b binds to C4b2a the complex forms C4b2aC3b, with C5 convertase activity. The AP is initiated by the generation of C3(H2O) after spontaneous hydrolysis of C3, or as a consequence of loop amplification triggered during LP and CP activation,. Since FB binds to C3(H2O), FB can be cleaved by FD in Ba and Bb. The Bb fragment remains bound to C3(H2O) forming the first C3 convertase of AP (C3(H2O)Bb), which cleaves C3 into C3a and C3b. C3b also has a binding site for FB, allowing cleavage by FD, resulting in the second C3 convertase of AP, C3bBb. Additional C3b molecules are able to bind to the C3bBb complex to form C3bBb3bn, which exerts C5 convertase activity. Both C5 convertases, LP/CL and AP, cleave the C5 component into C5a and C5b. Newly formed C5b reacts with C6 to form the stable C5b6 complex that recruits C7 resulting in a hydrophobic complex that targets the membrane (mC5b-7). Membrane insertion is initiated upon binding of C8 (C5b-8) and 12–18 copies of C9 polymerize to form the membrane attack complex (MAC) that induces lysis of target membranes. In addition, as a product of all pathways being activated, the small fragments C4a, C3a, and C5a are formed, which are important anaphylatoxins, attracting and activating inflammatory cells to the activation site, such as neutrophils, monocytes, macrophages, and dendritic cells.

The interaction of complement with *T. cruzi* is a principal step in the immediate immune response of the host against the parasite. However, it is important to consider that this interaction is dependent on the evolutive forms of the parasite. Experimental studies showed that complement can be activated by amastigote (Iida et al., 1989), epimastigote (Nogueira et al., 1975), and trypomastigote forms (Kipnis et al., 1985), but only non-infective epimastigote forms are susceptible to complement lysis. However, some strains of metacyclic trypomastigotes have been shown to be susceptible to complement-mediated killing *in vitro* (Cestari and Ramirez, 2010). Epimastigotes can be recognize by MBL, ficolins, C3 and C1q, but C3b and C4b deposition assays revealed that complement activation occurred mainly by the LP and AP in non-immune sera (Cestari and Ramirez, 2010). CP can also be activated through the binding of specific antibodies to epimastigote’s surface (Nogueira et al., 1975). As previously shown, AP can be activated by the spontaneous hydrolysis of C3; C3b is deposited on the epimastigote’s surface supporting efficient formation of AP C3 convertase (Joiner et al., 1986). Since all pathways are activated and C3 convertases are formed, C3 is cleaved into C3a and C3b with additional C3b molecules binding to C3 convertases, forming C5 convertases, which in turn cleave C5 into C5a and C5b. Then C5b recruits C6, C7, and C8 forming a C5b-8 complex where C9 molecules polymerize forming the MAC that induces the lysis of epimastigotes (**Figure 3**). Abrahamsohn and Dias da Silva (1977) demonstrated that epimastigotes treated with specific immune serum in the presence of splenic lymphocytes may be also killed *in vitro* by antibody dependent cell-mediated cytotoxicity.

During the differentiation cycle from non-infective/complement susceptible to infective/complement resistance forms, trypomastigotes acquire the ability to circumvent complement lysis by TcCRT, TcCRP, TcCRIT, gp58/68, and T-DAF molecules. At the first stages of *T. cruzi* infection (seconds after infection), complement can be initially activated by the LP and AP since both pathways do not depend on a specific antibody response (Cestari et al., 2013). Thus, trypomastigotes can immediately be targeted by complement after accessing the host bloodstream. A wide range of carbohydrates (such as GalNAc and GlcNAc) anchored by glycosylphosphatidylinositol in the outer leaflet of *T. cruzi* plasma membrane (Lederkremer and Bertello, 2001; Buscaglia et al., 2006) can be recognized by PAMP sensor molecules, such as MBL and ficolins (Cestari et al., 2009; Cestari and Ramirez, 2010) leading to the activation of MASPs. Then, the serine protease MASP-2 cleaves C2 and C4 generating LP C3 convertase formation that activates C3 to form C3b, contributing to the AP amplification loop with simultaneous LP and AP activation (Ricklin et al., 2010; Cestari et al., 2013). In addition, the activation of the AP can also take place spontaneously by hydrolysis of C3, which leads to the generation of C3 convertase and C3 cleavage. However, deposition of C3b on trypomastigotes may contribute to *T. cruzi* internalization by CR1 (van Lookeren et al., 2007). Both LP and AP can be continually activated, not only at the first stages of *T. cruzi* infection, but also during the course of chronic CD (Lidani et al., 2015). At a later stage of *T. cruzi* infection (days post-infection), CP becomes activated when anti-trypomastigote-specific IgM and IgG antibodies are produced allowing C1q binding and the activation of C1r and C1s serine proteases that cleave C4 and C2 forming the CP C3 convertase. Although during *T. cruzi* infection complement is activated by these three pathways, the process is interrupted at the C3 convertase level by complement regulatory proteins derived from trypomastigotes that cause abrogation of the terminal pathway and MAC formation. This evasion from complement lysis allows trypomastigote cell invasion and tissue spreading driving the infection toward chronic disease.

## Complement Evasion Strategies Used by *T. cruzi*

**FIGURE 4:**
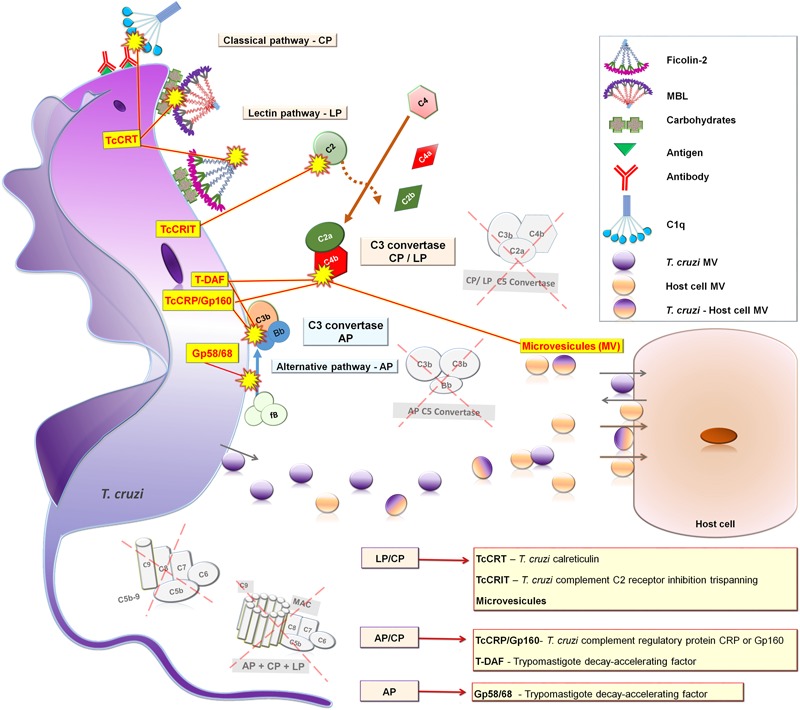
**Complement evasion by strategies of *T. cruzi* trypomastigotes forms.** TcCRT blocks CP and LP binding to C1q, MBL and Ficolin-2; T-DAF and Gp160/TcCRP block AP and CP C3 convertase assembly; TcCRIT blocks CP and LP binding to C2; gp58/68 blocks AP C3 convertase binding to factor B; and MV inhibit CP and LP C3 convertase assembly. AP, Alternative pathway; LP, Lectin pathway; and CP, Classical pathway.

The success of *T. cruzi* infection depends on a series of complex mechanisms that enable the parasite to evade the host immune response. In fact, *T. cruzi* employs a range of strategies to escape the effects of both innate and adaptive immunity. A crucial step occurs during the first seconds of infection when trypomastigotes needs to circumvent the harmful lytic attack of complement (Sacks and Sher, 2002). During differentiation from epimastigote to metacyclic trypomastigote forms inside the insect vector, the parasite undergoes a series of morphological and physiological changes that confers the capacity to evade the lytic effect of complement. The mechanism controlling this resistance mainly involves the expression of complement binding molecules on the trypomastigote’s surface, such as *T. cruzi* calreticulin (TcCRT) (Ferreira et al., 2004a; Valck et al., 2010; Sosoniuk et al., 2014), *T. cruzi* complement regulatory protein (TcCRP) or Gp160 (Norris et al., 1989; Norris and Schrimpf, 1994), *T. cruzi* complement C2 receptor inhibitor trispanning (TcCRIT) (Cestari et al., 2008), gp58/68 (Fischer et al., 1988) and T-DAF (Tambourgi et al., 1993). In addition, it has been shown that *T. cruzi* metacyclic trypomastigote forms induce membrane-derived vesicles (microvesicles) from host cells, which interact with C3 convertase, resulting in inhibition of complement activation and increased parasite survival, as well as eukaryotic cell invasion (Cestari et al., 2012). Moreover, it has been reported that microvesicles, derived from the host cell membrane and also secreted by *T. cruzi*, can fuse thereby increasing host cell invasion (Ramirez et al., 2016) (**Table 1**). The molecules and microvesicles involved in *T. cruzi* evasion from the complement system will be covered in more detail (**Figure 4**).

**Table 1:** Characteristics of the molecules involved in the evasion of *Trypanosoma cruzi* from the complement system.

Parasite molecule	Complement component	Complement pathway affected	Reference
TcCRT	Binds to MBL collagen-like domain	Lectin pathway	Ferreira et al., 2004b
	Binds to Ficolin-2 collagen-like domain	Lectin pathway	Sosoniuk et al., 2014
	Interacts with C1q collagen-like domain	Classical pathway	Ferreira et al., 2004b; Valck et al., 2010
T-DAF	Binds to C3b and C4b accelerating the dissociation of C3 convertase	Alternative, classical, andlectin (probably) pathways	Joiner et al., 1988; Rimoldi et al., 1988; Tambourgi et al., 1993
TcCRP/Gp160	Binds to C3b and C4b preventing assembly of C3 convertase	Alternative and classical Lectin (probably) pathways	Norris et al., 1991, 1989; Norris and Schrimpf, 1994; Norris, 1998
TcCRIT	Binds to C2 and prevent its cleavage by C1s and MASP2	Classical and lectin pathways	Cestari et al., 2008, 2009
gp58/68	Interacts with Factor B blocking its binding to C3b	Alternative pathway	Fischer et al., 1988
*T. cruzi*-induced membrane-derived vesicles from host cells or microvesicle (MV)	Binds to C3 convertase (C4b2a) on the *T. cruzi* surface, thereby inhibiting C3 cleavage	Classical and lectin pathway	Cestari et al., 2012; Ramirez et al., 2016

### *T. cruzi* Calreticulin (TcCRT)

*Trypanosoma cruzi* calreticulin (TcCRT) is a 45 kDa calcium-binding protein that is primarily expressed in the ER of infective trypomastigotes. Upon infection, TcCRT is translocated from the RE to the emerging area of the flagellum on the plasma membrane surface (Ferreira et al., 2004a,b; González et al., 2015). Interestingly, this area has been shown to be the initial contact with the host plasma membrane (González et al., 2015). TcCRT expressed on the *T. cruzi* trypomastigote’s surface is able to bind to host PRMs, similar to C1q, MBL (Ferreira et al., 2004b; Ramírez et al., 2011) and ficolins (Sosoniuk et al., 2014) interfering in the activation of the CP and LP. TcCRT also likely enhances the rate of the internalization of *T. cruzi* trypomastigotes by host cells (Ramírez et al., 2011, 2012).

*Trypanosoma cruzi* calreticulin was initially named Tc45 and described as a polypeptide present in lysates of epimastigotes and trypomastigotes. It was revealed to have an immunogenic role in the induction of specific antibodies in *T. cruzi*-infected mice (Ramos et al., 1991) and in human CD (Aguillón et al., 1997). Tc45 was subsequently characterized as *T. cruzi* calreticulin (TcCRT) as gene sequencing showed homology with calreticulin genes of other species (Aguillón et al., 2000). Later, TcCRT was found to exhibit a high homology with HuCRT, a multifunctional 46 kDa protein present predominantly in the ER and subcellular compartments, as well as in the plasma membrane (Ferreira et al., 2004a). Extracellular HuCRT has been reported to contribute to cell adhesion to the extracellular matrix and to facilitate the uptake of apoptotic and cancerous cells by phagocytes (Lu et al., 2015). The molecule may act as a receptor for the collagen-like domain of C1q and MBL, promoting phagocytosis through C1qR (Stuart et al., 1997). In fact, the role of C1q in the invasion of mononuclear phagocytes and fibroblasts by *T. cruzi* trypomastigotes has previously been demonstrated (Rimoldi et al., 1989). This parasite-derived molecule was later identified in infective trypomastigotes as TcCRT (Ferreira et al., 2004b) and its activity is considered one of the strategies that mediates *T. cruzi* uptake by mammalian phagocytic cells, mimicking the process of apoptotic cells ingestion (Ramírez et al., 2011).

In the early stages of infection, TcCRT is able to bind to the carbohydrate recognition domain of MBL resulting in abrogation of interaction with its natural ligands and the collagen domain of Ficolin-2, preventing C4 activation and impairing further activation of the LP (Ferreira et al., 2004b; Sosoniuk et al., 2014). This property however, is not shared by Ficolin-3 (Sosoniuk et al., 2014). During the late stages of *T. cruzi* infection the CP can be activated in the presence of specific *T. cruzi* antibodies, as mentioned earlier. Ferreira et al. (2004b) have demonstrated using hemolytic assays that recombinant TcCRT binds to C1q collagenous tails, impairing the activation of the CP. Subsequent studies showed that TcCRT competes with the (C1r-C1s)_2_ tetrameric complex for binding in the collagenous C1q tails and interferes in the capacity of C1s to activate C4 in a calcium-independent manner (Valck et al., 2010). In summary, TcCRT has been shown to be involved in the internalization of *T. cruzi* into mammalian cells, which increases infectivity. It is also known to be an important regulator of both the LP and CP.

### Trypomastigote Decay-Accelerating Factor (T-DAF)

The T-DAF is an 87–93-kDa glycoprotein present on the surface of metacyclic and tissue-culture-derived trypomastigote forms of *T. cruzi* that mimics the activity of the complement regulatory protein DAF (Tambourgi et al., 1993). T-DAF regulates the activation of the AP, CP, and probably LP, by interfering in the assembly of C3 convertases.

During *T. cruzi* infection, the three complement pathways are activated culminating with the formation of C3 convertase that cleaves the central component C3. The presence of molecules modulating the C3 convertase of the CP in trypomastigote but not epimastigote forms of *T. cruzi* surface was first described by Kipnis et al. (1986). It was subsequently demonstrated in a supernatant culture of trypomastigotes that these modulator molecules ranged between 86 and 155 kDa in size, that were enriched for proteins of 86–98 kDa absent in epimastigotes. They were shown to accelerate the decay of both the AP and CP C3 convertases *in vitro* by interfering in the binding of factor B with C3b, or in the formation of C4b2a, respectively. Because of the similar behavior to human complement regulatory DAF the authors called these modulators as analogous to DAF (Rimoldi et al., 1988). In the same year Joiner et al. (1988) characterized biochemically an 87–93 kDa factor produced by *T. cruzi* trypomastigote forms responsible for accelerating the decay of C3 convertases, which appeared to be an immunogenic glycoprotein immunoprecipitated from sera of patients chronically infected with *T. cruzi.* Later, Tambourgi et al. (1993) called this molecule analogous to DAF as T-DAF owing to its ability in inhibiting complement activation in a manner functionally similar to mammalian DAF. In addition, polyclonal and monoclonal antibodies against T-DAF were shown to inhibit T-DAF activity *in vitro*, validating its functional role (Tambourgi et al., 1993). The authors also showed that T-DAF shared 40% similarity with a portion of the DNA coding region for human DAF, and that anti-T-DAF-specific antibodies were present in up to 96% of patients with both acute and chronic CD (Tambourgi et al., 1995). In summary, T-DAF either accelerates the dissociation or assembly efficiency of C3 convertases potentially affecting the AP, CP (Tambourgi et al., 1993), and probably LP. Both soluble and surface T-DAFs are essential for the escape of *T. cruzi* from complement activation and lytic effects as well as the development of infection.

### *Trypanosoma cruzi* Complement Regulatory Protein (TcCRP)

*Trypanosoma cruzi* complement regulatory protein (TcCRP), also called Gp160, is a 160 kDa glycoprotein anchored into trypomastigote membranes (Norris et al., 1989) via glycosylphosphatidylinositol linkage (Norris and Schrimpf, 1994). It is also spontaneously shed in culture from trypomastigotes (Norris et al., 1991). Both membrane and soluble forms of TcCRP are able to bind to C3b and C4b inhibiting the formation of the AP and CP C3 convertases (Norris et al., 1991; Norris and Schrimpf, 1994), and probably LP C3 convertase as well.

*Trypanosoma cruzi* complement regulatory protein was initially purified and partially characterized by Norris et al. (1989) as Gp160 because of its molecular weight, and was found to be expressed in membrane extracts of both metacyclic and tissue-culture-derived *T. cruzi* trypomastigotes, but absent in insect epimastigotes or intracellular amastigotes. Norris and Schrimpf (1994) purified and characterized the membrane form of TcCRP having the glycolipid anchor attached which presented a molecular mass of 185 kDa. The conversion of the 185-kDa membrane form to the 160-kDa form was suggested to be the result of cleavage by endogenous phospholipase C. Both soluble (160 kDa) and membrane TcCRP forms (185 kDa) are able to bind C3b and C4b preventing the assembly of proteolytically active C3 convertase inhibiting both AP and CP activation (Norris et al., 1991; Norris and Schrimpf, 1994). It was then demonstrated that the TcCRP had similar activity to human CRP and DAF, being considered a member of the C3/C4 binding family of complement regulatory proteins, which provided infectious trypomastigotes another mean of evading the harmful effects of complement (Norris et al., 1991). Since TcCRP binds C4b, LP activation could be also affected at the same level. Moreover, anti-TcCRP lytic antibodies were present in the sera of *T. cruzi*-infected patients. Interesting, in the presence of anti-TcCRP antibodies the interaction between TcCRP and C3b was blocked allowing the AP C3 convertase assembly and parasite lysis (Norris et al., 1991).

Norris (1996) demonstrated that following the binding to C3b, TcCRP was released from the *T. cruzi* trypomastigote membrane by proteolytic cleavage, and these findings represented a new alternative mechanism of *T. cruzi* in evading complement activation. Interestingly, epimastigote forms transfected with a cDNA encoding full-length recombinant TcCRP were protected from complement-mediated lysis, confirming the role of TcCRP as a complement resistance factor of *T. cruzi* trypomastigotes (Norris et al., 1997; Norris, 1998). Recently, Henrique et al. (2016) demonstrated that surface expression of TcCRP differs among parasite strains, with a tendency of higher expression levels in the most virulent *T. cruzi* strains. In summary, TcCRP is directly involved in the evasion of *T. cruzi* from complement lysis by binding to C3b and C4b and consequently inhibiting both the AP, CP, and probably LP.

### *T. cruzi* Complement C2 Receptor Inhibitor Trispanning (TcCRIT)

*Trypanosoma cruz*i complement C2 receptor inhibitor trispanning (TcCRIT) is a 32 kDa transmembrane protein that presents a sequence homology with the C4 beta-chain, the binding site of C2. Thus, TcCRIT inhibits the cleavage of C2 by C1s or MASP-2 and consequently prevents C3 convertase formation by competing with C4 (Inal, 1999; Inal and Schifferli, 2002; Cestari et al., 2012). TcCRIT is mainly expressed in complement resistant trypomastigote forms of *T. cruzi* regulating the activation of both the CP and LP (Inal, 1999; Inal et al., 2005).

The complement C2 receptor inhibitor trispanning (CRIT) was first described in the tegument of *Schistosoma haematobium* and subsequently *S. mansoni* (Inal, 1999; Inal and Sim, 2000) as a phosphorylated tyrosine molecule named TOR that inhibited C2 cleavage by C1s inhibiting the CP activation. It was then characterized as a novel complement regulator, and named CRIT (Inal and Sim, 2000). *S. haematobium* TOR (Sh-TOR) protein sequencing showed a long cytoplasmic tail with several consensus phosphorylation sites for enzymes, such as tyrosine kinases, characteristically associated with membrane receptors (Inal, 1999). Later, it was demonstrated that the Sh-TOR synthetic peptide pre-incubated with C2 inhibited the CP activation both *in vitro* (Inal and Schifferli, 2002) and *in vivo* (Inal et al., 2003) by competing with C4b (which presents some sequence identity to the first extracellular domain of Sh-TOR) (Inal and Sim, 2000). CRIT is highly conserved in *Schistosoma* species, *T. cruzi* strains, and mammals (Inal, 1999, 2005). Interestingly, both the *Trypanosoma* parasite and its human host share a receptor for C2, with a complement regulatory function (Inal et al., 2005).

Complement C2 receptor inhibitor trispanning protein was later shown to be expressed in the infective stage of *T. cruzi* preventing the lytic activity of NHS. In addition, overexpression of TcCRIT in transgenic epimastigotes increased the resistance to complement-mediated killing in the presence of non-immune NHS, blocking both the CP and LP. However, when epimastigotes were treated with exogenous C2 the complement activity was restored (Cestari et al., 2008). Although metacyclic trypomastigotes and epimastigotes differ regarding complement resistance, MBL, Ficolin-2 and Ficolin-3 are able to bind to glycosylated proteins on the surface of both parasites forms. However, only metacyclic trypomastigotes were shown to resist complement killing due to high expression levels of TcCRIT. TcCRIT circumvent LP activation through binding to C2, inhibiting its cleavage by MASP-2 and C3 convertase formation (Cestari et al., 2009). In summary, TcCRIT interferes in the activation of the LP and CP by binding to C2 and preventing its cleavage by the serine proteases C1s and MASP2, thereby evading both initial branches of complement activation. This leads to resistance against complement-mediated cell lysis, allowing *T. cruzi* survival and cell invasion.

### *T. cruzi* Complement Regulatory gp58/68

The *T. cruzi* complement regulatory gp58/68 is a glycoprotein of an apparent molecular weight of 58 kDa (non-reduced) and 68 kDa (reduced) (Fischer et al., 1988) that is expressed on the parasite surface or can be released by trypomastigotes in culture (Ouaissi et al., 1988; Velge et al., 1988). It is part of the *T. cruzi* fibronectin/collagen receptor which consists of two molecules of 80–85 kDa and 58–68 kDa with an important role in the attachment of trypomastigote forms to mammalian cells (Ouaissi et al., 1984, 1986; Velge et al., 1988). Gp58/68 acts as a *T. cruzi* complement regulatory protein shown to inhibit the formation of cell-bound and fluid-phase AP C3 convertases (Fischer et al., 1988).

Gp58/68 was first identified in studies on the biological function of *T. cruzi* fibronectin receptors (TcFnR) by Ouaissi et al. (1984). Human fibronectin purified from blood were shown to bind specifically to *T. cruzi* trypomastigotes and to be involved in cell-parasite interaction. Anti-fibronectin antibodies were able to eradicate *in vitro* fibroblast infection by *T. cruzi* trypomastigotes (Ouaissi et al., 1984). The isolation and functional characterization of TcFnR were later achieved using immunoprecipitation assays, which identified the 85 kDa protein. Both affinity-purified TcFnR and anti-TcFnR antibodies exerted an inhibitory effect in the infection of fibroblasts by *T. cruzi* trypomastigotes in culture (Ouaissi et al., 1986). Subsequent studies characterized the other part of the fibronectin/collagen receptor, the collagen receptor, and showed that the same *T. cruzi* receptor binds to the host fibronectin and/or collagen, and that both the 80–85 kDa and 58/68 kDa glycoproteins are part of the same receptor (Velge et al., 1988). In the same year, the gp58/68 was purified by affinity chromatography from lysate of both culture and peripheral blood *T. cruzi* trypomastigotes and named according to its molecular weight (Fischer et al., 1988). This *T. cruzi* glycoprotein was able to inhibit the formation of cell-bound AP C3 convertase by preventing the initial association of FB with surface fixed C3b in a dose dependent manner. Moreover, gp58/68 was also shown to restrict the formation of fluid-phase AP C3 convertase by the consumption of fluid-phase FB (Fischer et al., 1988). In summary, gp58/68 is part of a fibronectin/collagen receptor of *T. cruzi* that has an important role in the interaction of *T. cruzi* with mammalian cells and confers the parasite’s ability to evade AP complement activation by inhibiting FB/C3b interaction.

### Host and *T. cruzi* Microvesicles

Microvesicles (MVs) are 100–1000 nm vesicles originating from the plasma membrane and are released by a large number of cells from the blood, immune system, epithelial and endothelial tissues, among others (Evans-Osses et al., 2013). MVs are involved in intercellular communication owing to their capacity to transfer proteins, lipids, and nucleic acids, thereby influencing various physiological and pathological functions of both the recipient and parent cell (Yáñez-Mó et al., 2015). MVs are known to be released by different pathogens such as bacteria, fungus, and parasites, including *T. cruzi* (Silveira et al., 1979; Gonçalves et al., 1991; Geiger et al., 2010), which may carry virulence factors to host cells promoting the dissemination of the pathogen (Deatheragea and Cooksona, 2012; Barteneva et al., 2013). Host mammalian cells infected with *T. cruzi* release MVs that interfere in CP and LP C3 convertases assembly on the parasite’s surface, leading to the inhibition of its catalytic activity (Cestari et al., 2012) and consequently eliminating complement activation. Recent findings have demonstrated that interaction between *T. cruzi* trypomastigote and epimastigote forms with host cells also can induce MVs formation (Ramirez et al., 2016).

New insight into the mechanisms of complement immune evasion by *T. cruzi* were gain when Cestari et al. (2012) reported secretion of host MVs induced by parasitic infection. At the beginning of infection, metacyclic trypomastigotes induced the release of MVs from immune cells, such as lymphocytes, monocytes, and macrophages in a calcium-dependent process (Cestari et al., 2012). Under experimental conditions MVs were shown to strongly inhibit C3b deposition. However, C4b deposition was not significantly inhibited. These findings suggest that MVs interfered on complement at C3 level. In addition, MVs were shown to bind LP and CP C3 convertase complexes on the surface of *T. cruzi* inhibiting complement-mediated lysis and favoring the invasion of host cells. Moreover, C1q, Ficolin-2, and Ficolin-3 were also found to bind to MVs but did not impair parasite recognition by these PRMs. It has also been shown that MVs derived from lymphocytes and monocytes carry TGF-β, an important cytokine that enhances *T. cruzi* cell invasion and protects the parasite against complement-mediated lysis (Cestari et al., 2012). Recently, the release of MVs derived from infective (metacyclic trypomastigote and tissue-culture-derived trypomastigote) and non-infective (epimastigote) parasites, and their interaction with host cells was demonstrated. In addition, infective and non-infective *T. cruzi* forms were shown to induce different levels of MV release from host cells. Moreover, the fusion of MVs derived from both host cell and parasite was demonstrated, and this phenomenon appears to facilitate contact between *T. cruzi* and host cell plasma membranes, and probably membrane fusion. Thus, MVs released during interaction of the parasite with host cells were able to increase host cell invasion by metacyclic trypomastigotes (Ramirez et al., 2016). In summary, interaction between *T. cruzi* and host cells prompt the release of MVs from both parasite and host cells and this phenomenon may contribute to evasion of CP and LP complement activation and to increase host cell infection. Thus, both host and parasite MVs probably have a potential immunomodulatory effect in the pathogenesis of *T. cruzi* infection.

## Immunomodulatory Perspectives

Although it has been suggested that the effectiveness of the etiological treatment for CD is inversely proportional to the duration of *T. cruzi* infection, the success of trypanosomatid drugs in preventing clinical progression to symptomatic forms is still controversial. Considering that *T. cruzi* makes use of several strategies to evade the host immune system in order to establish the infection, these molecules involved in evasion mechanisms could be interesting therapeutic targets to be explored in the context of prevention and treatment of CD. Immunomodulatory therapies could be based on the use of antibodies against overexpressed *T. cruzi* molecules, such as TcCRT, T-DAF, TcCRP, TcCRIT, and gp58/68. For instance, *T. cruzi* complement regulatory proteins, such as TcCRP, T-DAF, and gp58/68, may also be targets of anti-*T. cruzi* lytic antibodies (as reviewed by Krautz et al., 2000) and could be used as indicators of drug efficacy in *T. cruzi* infection and parasite clearance, as observed by anti-TcCRP and anti-T-DAF detected in sera of CD patients (Norris et al., 1994; Tambourgi et al., 1995). In addition, the association of MBL deficiency with protection against the development and progression of chronic CD cardiomyopathy (Luz et al., 2016) highlighted a potential marker for disease progression. Moreover, an interesting therapeutic approach to control the early stage of *T. cruzi* infection in patients with MBL deficiency and defects in LP activation may be the restitution of MBL or other PRMs involved in complement activation. Thus, new investigations are needed to explore which *T. cruzi* evasion molecules could be potential immunotherapeutic targets that could contribute to change the pathophysiological progression of this neglected disease.

## Conclusion

The current knowledge on complement evasion strategies used by *T. cruzi* highlights the importance of the LP, AP, and CP as crucial components in the first line of defense against this parasitic infection. However, *T. cruzi* infective forms are able to regulate and inhibit the complement activation early in the proteolytic cascade by expressed and/or released regulator molecules thereby circumventing complement’s harmful effects. Thus, complement becomes prey of *T. cruzi* and has a bad day. Understanding these complement evasion strategies is crucial for the development of innovative strategies in the battle against *T. cruzi* infection and may pave the way for novel immunotherapies.

## Author Contributions

KL, LB, and AA participated in the design and writing of the manuscript. IdM-R: participated in the design, coordination, and manuscript writing. KL developed the graphic design of all figures.

## Conflict of Interest Statement

The authors declare that the research was conducted in the absence of any commercial or financial relationships that could be construed as a potential conflict of interest. The reviewer WDDS and handling Editor declared their shared affiliation, and the handling Editor states that the process nevertheless met the standards of a fair and objective review.

## Abbreviations

AP: alternative pathway
C1-INH: C1 esterase inhibitor
C1qR: C1q receptor
C4BP: C4b-binding protein
CD: chagas disease
CD46: membrane cofactor protein
CD59: inhibitor membrane attack complex-inhibitory protein
CP: classical pathway
CL-K1: collectin -11
CRIT: complement C2 receptor inhibitor trispanning
CR1: complement receptor 1
DAF: decay accelerating factor or CD55
ELISA: enzyme-linked immunosorbent assay
ER: endoplasmic reticulum
FB: factor B
FD: factor D
GlaNAc: *N*-acetylgalactosamine
GlcNAc: *N*-acetyl-D-glucosamine
HuCRT: human calreticulin
IFA: immunofluorescence antibody test
LP: lectin pathway
MAC: membrane attack complex
MASP-1: MBL-associated serine proteases - 1
MASP-2: MBL-associated serine proteases - 2
MBL: mannose-binding lectin
MV: microvesicles
NHS: normal human serum
PAMPs: pathogen-associated molecular patterns
PCR: polymerase chain reaction
PRMs: pattern-recognition molecules
Sh-TOR: *Schistosoma haematobium*
*T. cruzi*: *Trypanosoma cruzi*
TcCRT: *Trypanosoma cruzi* calreticulin
TcCRIT: *Trypanosoma cruzi* complement C2 receptor inhibitor trispanning
TcCRP: *Trypanosoma cruzi* complement regulatory protein or Gp160
T-DAF: trypomastigote decay-accelerating factor
TOR: trispanning orphan receptor
